# The clinicopathological features of second primary cancer in patients with prior breast cancer

**DOI:** 10.1097/MD.0000000000006675

**Published:** 2017-04-21

**Authors:** YiHui Liu, ChunHui Dong, Ling Chen

**Affiliations:** a Department of Oncology, The First Affiliated Hospital of Xi’an JiaoTong University, Xi’an, Shaanxi; b Department of Oncology, The Ninth Hospital of Xi’an, Xi’an, P.R. China.

**Keywords:** breast cancer, clinicopathological features, multiple primary cancers, second primary cancer

## Abstract

Nowadays, the risk of developing second primary cancers among women diagnosed with prior breast cancer represents a public health issue worldwide.

Twenty-eight cases of the primary breast cancer with the multiple primary cancers (MPC) between 2008 and 2015 at our hospital were retrospectively analyzed in regards to age of patients, family history, interval time of the 2 cancers, and survival time of these patients.

A total 28 cases were analyzed, at the mean age of 44.57 years at the diagnosis of the first primary cancer. The most common primary cancer in these breast cancer patients was contralateral breast cancer. Of 28 patients with breast cancer, 16 developed a second malignant tumor of the opposite breast, there were no significant difference both median age at first breast cancer and second breast cancer (*P* > .05). The difference of interval time of 2 cancers also had no statistical significance. There was no statistically significant difference in overall survival between the bilateral primary breast cancers (BPBC) group and the group of breast cancer patients who diagnosed with another cancer (*P* > .05). If we grouped patients age of diagnosed with the first cancer (<45, ≥45 years), no statistical different between 2 groups (*P* > .05). However, the survival time with positive-node patients was lower than in patients with node-negative, the difference had a notable significant difference (*P* < .01). And there are 3 cases had a positive family history for malignant tumor in the form of first-degree relative.

Multiple primary carcinoma in patients with prior breast cancer is not the influencing factor of prognosis. It is crucial to detect, diagnose, and treat cancers at their early stage for improving the cure rate of cancer and the survival rate of patients.

## 1 Introduction

According to the statistic analyze of the cancer incidence and mortality, breast cancer is the most common malignancy diagnosed in female.^[1]^ In the world, it was estimated that new cancer cases and cancer deaths were 1.3 million and 327,000 every year.^[2]^ In China, reported in 2012, about 273,000 women develop new malignant carcinoma of the breast cancer, meanwhile, there were 62,000 breast deaths, respectively.^[3]^ With the development of the medical technology, surgical treatment and adjuvant therapy increased the chances of survive from breast cancer. Early detection, early diagnosed, and early adequate treatment may have led to prolonged survival and improved quality of life for breast cancer patients. Nevertheless, the long-term health of these patients will become a significant public health problem because the possibility of developing second primary cancers may be on the rise.^[4]^

Multiple primary cancers (MPC) is generally defined according to the criteria of Warren and Gates,^[5]^ namely: the tumors must be clearly malignant on histological examination; the tumors must found in the different parts and had its own unique pathologic morphology; and the possibility that the second tumor represents a metastasis must be exclude. Some cancer registry studies evaluated that the risk of second primary cancer among women who diagnosed with prior primary breast cancer is higher than general population.^[6–8]^ Subsequent cancers after a breast cancer could be attributed either to common risk factors predisposing to both the first and second cancer, such as living environment, genetic predisposition, treatment-related side effects, or the other identified risk factors.^[9]^ Although there are several studies devoted to the cause, clinical profile and others of MPC, the characteristics are still unclear. Therefore, in this study, we analysis general features of multiple primary cancers patients after diagnosed with a primary breast cancer in our hospital, investigate the clinical profile and outcome of them.

## 2 Materials and methods

### 2.1 Patients and lesions

A research of our database identified 28 patients with breast cancers who had diagnosed with MPC at The First Affiliated Hospital of Xi’an JiaoTong University between January 2008 and December 2015. Among them, 16 patients diagnosed with bilateral primary breast cancers (BPBC). We excluded women who were not eligible for our criterion. The following conditions were our inclusion criteria: all the patients had to conform to the postoperative pathologic diagnosis and they had to diagnose the breast cancer with the first malignant tumor, the 2 tumors had independent pathological morphology, and it had to exclude possibility of recurrence and metastasis. Finally, 28 patients with breast cancers sets constituted our study population. Patients with BPBC were defined as those with different pathological patterns of contralateral breast after their initial diagnoses of breast cancer. For synchronous or metachronous cancers associated with breast cancer, divided at 6 months, when 2 primary cancers were detected with 6 months, they were defined synchronous. Similarly, if the 2 primary tumors were detected more than 6 months, they were considered metachronous primary cancers.

### 2.2 Data collection

All patients with MPC were histologically diagnosed by postoperative pathologic diagnosis. All clinicopathologic data were obtained from our database. The clinical data collected included the patients’ age at diagnosis, interval time of diagnosed the 2 cancers, treatment of the first cancer, position of the second tumorigenesis, the first menstruation time of these patients and a personal or first-degree family history of breast cancer.

The study was approved by the Xi’an JiaoTong University First Affiliated Hospital, Xi’an, China.

### 2.3 Statistical analysis

For the age and the interval time of 2 cancers comparison, we used independent sample *t* test, the age included the age of diagnosed with first cancer and the second cancer had diagnosis. Meanwhile, we used Kaplan–Meier method to make survival analysis. *P* < .05 indicates the difference was statistically significant. All statistics analyses were performed using SPSS software.

## 3 Results

### 3.1 Clinicopathologic factors associated with specific kinds of tumor

We had analyzed 28 patients, divided into 2 groups, included 16 (57.14%) BPBC and 12 (42.86%) one-side breast cancer patients with another primary cancer. Among 28 patients with breast cancer, 4 (14.29%) were diagnosed with synchronous primary cancer, 24 (85.71%) were diagnosed with metachronous primary cancer. And the pathological of first breast cancer was invasive ductal carcinoma (78.57%), invasive lobular carcinoma (14.29%), lobular carcinoma in situ (3.57%), and mucinous carcinoma (3.57%).

These patients were diagnosed with breast cancer (Table 1), the most common primary cancer in these breast cancer patients was contralateral breast cancer (57.14%), followed by thyroid cancer (17.86%), endometrial cancer (10.71%), cervical cancer (7.14%), rectal cancer (3.57%), and esophageal cancer (3.57%). The mean age of these patients was 44.57 years between the ages of 27 and 72 years, thyroid cancer had the youngest age of 36.6 years, while endometrial cancer had the oldest age of 49.33 years. The mean interval time from the first cancer diagnosis to the second cancer diagnosis was 83.89 months, and the mean age of the second cancer diagnosis was 51.57 years. There were no significant difference both median age at first breast cancer (*t* = 0.728, *P* = .787) and second breast cancer (*t* = 0.618, *P* = .474). The difference of interval time of 2 cancer also had no statistical significance (*t* = 0.273, *P* = .787).

**Table 1:**
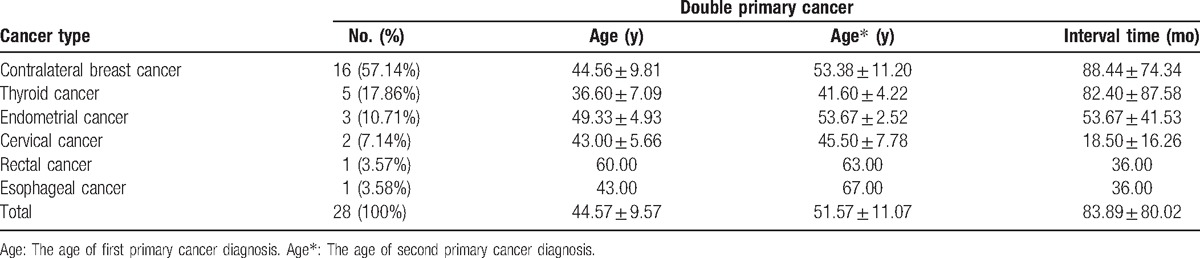
Cancer type, mean age, and interval time distribution in breast cancer patients.

### 3.2 Tumor therapy

The treatment of 28 cases is reported (Table 2). All of these patients had underwent resection, 9 (32.14%) of 28 underwent radical mastectomy and 19 (67.86%) underwent modified radical mastectomy. Sixteen (57.14%) of 28 patients received chemotherapy, 12 (42.86%) of 28 received radio therapy, and 9 (32.14%) received hormone therapy. Besides, 3 (10.71%) cases had a positive family history for malignant tumor in the form of first-degree relative.

**Table 2:**
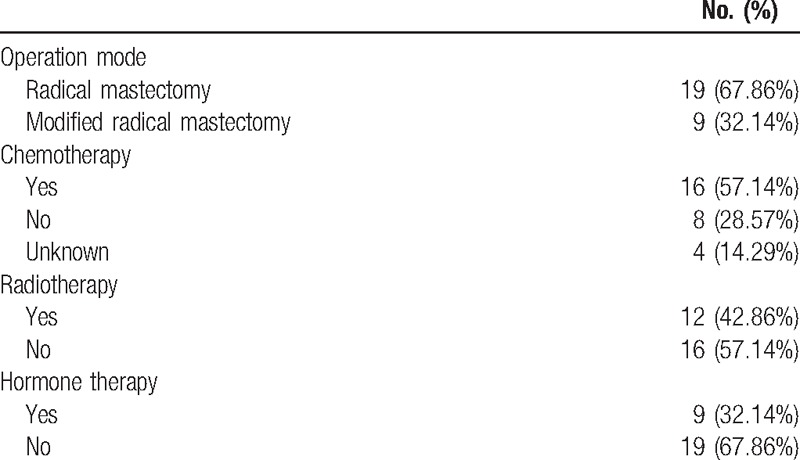
Treatment of the first breast cancer.

### 3.3 Results of followed-up patients

**Figure 1:**
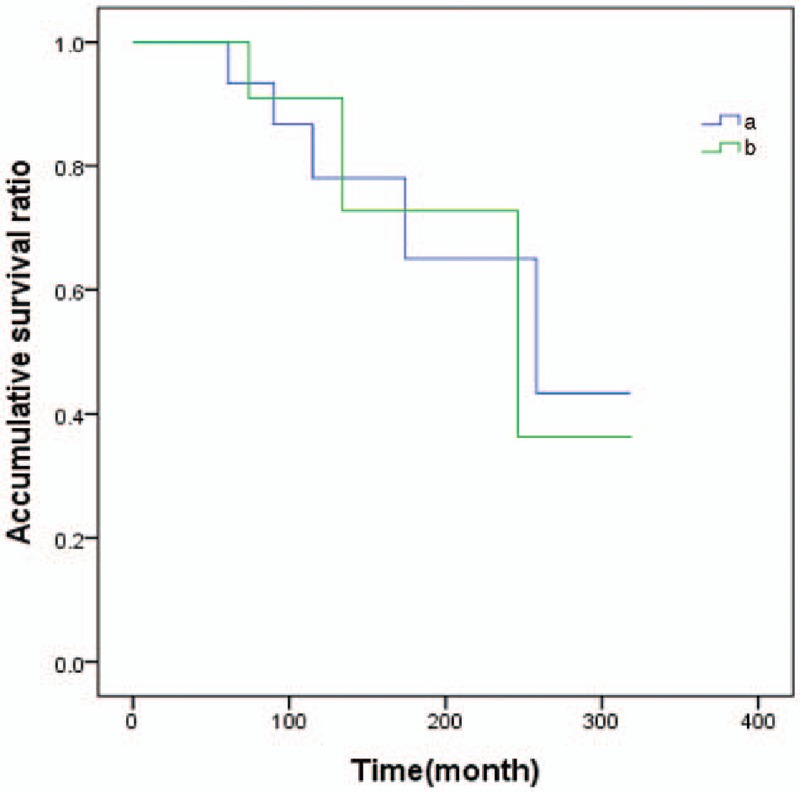
The survival analysis curve of second primary cancer in patients with prior breast cancer by type. Note: (a) bilateral primary breast cancers (BPBC); (b) breast cancer patients who diagnosed with another second cancer.

**Figure 2:**
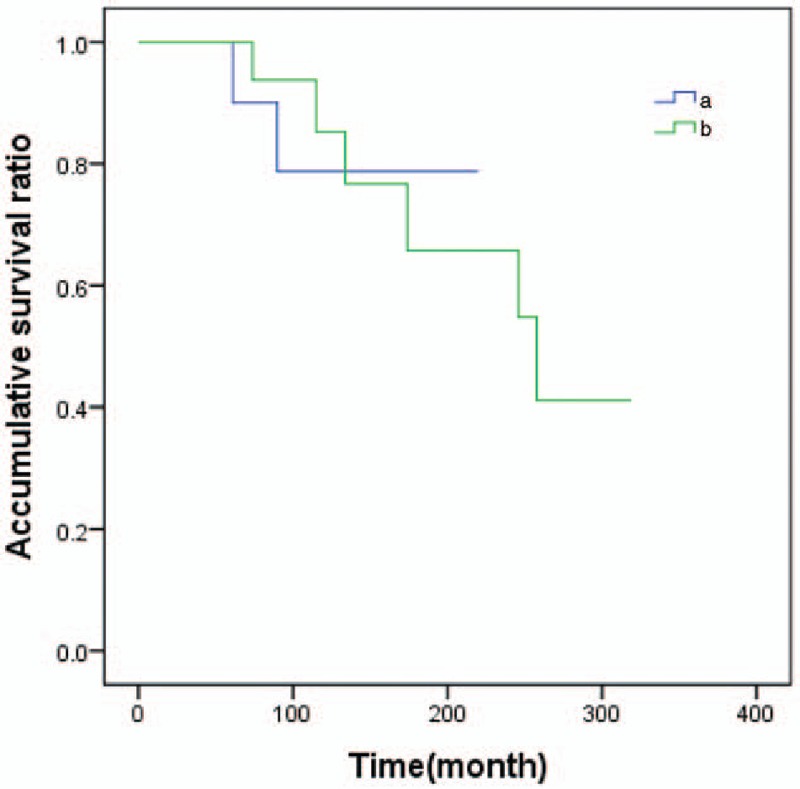
The survival analysis curve of second primary cancer in patients with prior breast cancer by age. Note: (a) Age <45 years patients when diagnosed the first breast cancer; (b) age ≥45 years patients when diagnosed with the first breast cancer.

**Figure 3:**
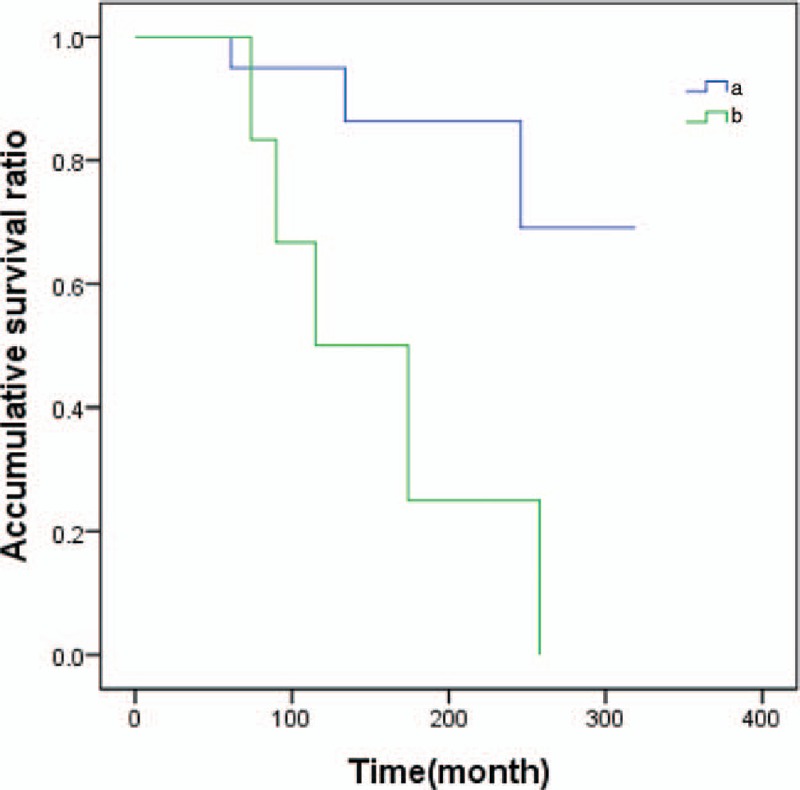
The survival analysis curve of second primary cancer in patients with prior breast cancer by axillary lymph node status. Note: (a) Patients with negative axillary lymph nodes; (b) patients with positive axillary lymph nodes.

All patients were followed-up for the composite outcome of tumor recurrence, distant metastasis, and death. When the age of diagnosed with the first breast cancer was used to calculate, the median follow-up time was 130 months (5–319 months). Three patients were found local recurrence, 1 patient was found distant metastasis, and 4 patients died. Local recurrence, distant metastasis and death, as the ultimate outcome, carried on survival analysis used Kaplan–Meier method (Figs. 1–3).

There was no statistically significant difference in overall survival between the BPBC group and the group of breast cancer patients who diagnosed with another cancer (*χ*^2^ = 0.007, *P* = .926 > .05). If we grouped patients age of diagnosed with the first cancer (<45, ≥45 years), no statistical different between 2 groups (*χ*^2^ = 0.004, *P* = .950 > .05). The state of axillary lymph node was the influence of factor patients’ survival time, the survival time with positive-node patients was lower than in patients with node-negative, the difference had a notable significant difference (*χ*^2^ = 7.358, *P* = .007 < .01).

## 4 Discussion

Since MPC was first described by Billroth in 1869, it has been attracted more attention among people. This issue may be particularly relevant with the prolongation of the survival time of prior breast cancer, the incidence of second primary cancers increased. Thus, it is necessary to pay close attention to the risk of tumors latency different period. At present, the treatment of malignant carcinoma main includes surgery, chemotherapy, radiotherapy, endocrine therapy, and immunotherapy. In this study, all of these patients had undergone surgery, only a small proportion of patients received postoperative adjuvant therapy.

To the best of our knowledge, the incidence of second cancer after diagnosis or treatment of the first cancer has been risen continuously. Multiple primary cancers seem to be frequently increasing, lots of studies shown a high risk of second cancer in cancer patients. For example, a population-based study in North Portugal reported cancer survivors had higher incident rates of cancer than the general population.^[10]^ At present, its etiology and pathogenesis are not very clear, this may be related to hereditary factor, environmental factor, and adjuvant therapy of the first cancer. It was reported women with 4 cancers in 1 breast are 3 times more likely to develop a primary cancer in other breast.^[11]^ Schaapveld et al^[12]^ observed that chemotherapy was associated with a decreased hazard for all secondary nonbreast cancers combined, as well as colon and lung cancer, in patients younger than 50 years at breast cancer diagnosis. Also, several studies also have reported increased risks of second primary malignancies among breast cancer patients treated with postoperative radiotherapy.^[13,14]^ Grantzau et al^[15]^ analyzed the long-term risk of second solid nonbreast cancer of 46,176 patients treated for early breast cancer and concluded the radiotherapy treated breast cancer patients have a small but significantly excess risk of second cancers. Besides, research had shown tamoxifen may enable estrogen biosynthesis and metabolism changed, resulted in endometrial thickening and malignant transformation.^[16]^ Due to the small sample and incomplete information of these cases, the correlation between therapy and outcome of second primary cancer was not clear, there was still some limitations in this study. Next, we will collect more clinical cases of the therapy of malignant breast cancer and outcome of second primary cancers, analyze whether or not there is relationship between them.

In this study, the pathological of first breast cancer was invasive ductal carcinoma, invasive lobular carcinoma, lobular carcinoma in situ, and mucinous carcinoma. It is significant to analyze the relationship between different subtypes of breast cancer and the incidence of second primary cancers. As the second commonest histologic type of breast cancer after invasive ductal carcinoma,^[17–20]^ invasive lobular carcinoma has the diffuseness of the tumor, multifocality, and occult involvement of the contralateral breast. Due to the limited number of cases, the trend is not obvious in this study. There is a need for a follow-up study of the association between this subtype and second primary cancers.

It is reported that the first primary cancer predilection site of MPC was throat, bladder, and breast, the most common second cancer was lung, followed by breast cancer.^[21]^ In this study, we collected 28 patients with MPC that the first primary cancer was breast cancer. The most common second primary cancer in this study was contralateral breast cancer, followed by thyroid cancer, endometrial cancer, cervix cancer, rectal cancer, and esophagus cancer. Young and Suk^[22]^ studied the incidence of endometrial, stomach, and thyroid cancer increased significantly after treatment with primary breast cancer was observed in Korea. The difference of result, probably due to type of treatment, geographic position, or the small number of cases.

According to reports in the literature, the most intervals between 2 tumors of MPC were in the period of 5 years.^[23,24]^ In this study, the mean interval time from the first cancer diagnosis to the second cancer diagnosis was 83.89 months. Three patients had positive family history. Studies have shown that molecular genetics research of cancer genes provides evidence for the occurrence of cancer, such as breast and ovarian cancers, which is associated with mutations in autosomal dominant genes.^[25]^

Whether calculated the survival time as diagnosed with first cancer or second cancer is a debatable point. Our study adopted the age of first breast cancer to calculate survival time is based on analyses these patients as a group of special population. It is more accorded with their clinical characteristics. In this study, we find the status of axillary lymph node is an influence factor of patients’ survival time.

It is crucial to detect, diagnose, and treat cancers at their early stage for improving the cure rate of cancer and the survival rate of patients. The key to the early treatment of MPC is figure out the pathology type of the second cancer, and then eradicate it as much as possible. Whether to the patients preliminarily diagnosed with cancer or revisiting patients, there are 3 points that we should follow up as a clinician. Firstly, we should pay more attention to the danger of synchronous or metachronous multiple primary cancers. Then, the regular follow-up is necessary. Last, active treatment to the second cancer is the key to prolong survival time and improve patients’ quality of life.

## 5 Conclusions

The most common second primary cancer in this study was contralateral breast cancer, followed by thyroid cancer, endometrial cancer, cervix cancer, rectal cancer, and esophagus cancer. Multiple primary carcinoma in patients with prior breast cancer is not the influencing factor of prognosis. It is crucial to detect, diagnose, and treat cancers at their early stage for improving the cure rate of cancer and the survival rate of patients.
